# Analysis of the evolutionary process of traditional village spatial pattern: The case of Gaoyi village in Western Hunan, China

**DOI:** 10.1371/journal.pone.0309814

**Published:** 2024-09-06

**Authors:** Yunyuan Deng, Wenlong Zhou, Xiangxiang Fu, Yao Yao

**Affiliations:** 1 College of Geography and Tourism, Hengyang Normal University, Hengyang, Hunan, China; 2 National-Local Joint Engineering Laboratory on Digital Preservation and Innovative Technologies for the Culture of Traditional Villages and Towns, Hengyang, Hunan, China; 3 Cooperative Innovation Center for Digitalization of Cultural Heritage in Traditional Villages and Towns, Hengyang, Hunan, China; 4 School of Geographic Sciences, East China Normal University, Shanghai, China; Northeastern University (Shenyang China), CHINA

## Abstract

The examination of the characteristic law of traditional village transformation over time represents a vital nexus in cultural heritage preservation and the transmission of vernacular culture. Historical event points were used to augment village development information, facilitating the restoration of the village’s historical pattern. Geographical analysis methods, including Standard Deviation Ellipse Analysis (SDSEA), Nearest Neighbor Analysis (NNA), and Source-Destination Analysis (SDA), were employed to explore the characteristics of the village’s geographical center of gravity, changes in concentration and dispersion, and functional transfer. The stepwise progression of the village’s evolution was investigated, as well as the mechanism of residents’ behavior during the evolution process. The results reveal: 1) The spatial evolution of the settlement shows a trend of agglomeration. As time passes, the center of gravity of each functional space gradually converges, and the average distance between elements decreases, resulting in a shift from a dispersed to a clustered distribution. 2) The village space changes from simple to complex due to the conduct of the villagers. Residential behaviors promote the establishment of residential space and the development of public and commercial space. The usage, abandonment, and functional transitions that occur inside the space cause functional zones to nest with each other, resulting in a more intricate spatial structure. 3) Both the degree of change and the preservation of the village’s functional space show an increasing trend, indicating that the protection of the built space and the expansion of the unbuilt space occur simultaneously. This represents a developmental trend that is consistent with the social surroundings and the villagers’ ambitions.

## 1. Introduction

Traditional villages embody the cumulative actions of villagers, showcasing their long-standing wisdom and ability to adapt over many decades. An analysis of the spatial development of traditional villages allows us to understand their spatial organization and uncover the fundamental functioning dynamics of village spaces. Studying the development of traditional village spaces is a crucial measure in facilitating efficient governance and construction of these areas. This study is particularly significant given the current climate of heightened environmental awareness.

The study focuses on summarizing the characteristics of the spatial layout of traditional villages across time, in order to understand their evolution. Consequently, its associated research includes the discoveries of conventional research on spatial patterns in villages. Examples of activities include the disassembly of the internal components of villages and the division of functional spaces [1,2], the classification and assessment of the spatial features of villages [3,4], and the evaluation of the current spatial development status and recommendations [5]. Over time, this type of research has progressively broadened in two dimensions. One analytical perspective involves establishing a logical connection between the process of spatial form change and the changes in specific elements such as blood relationship and structural characteristics [6–8]. This perspective aims to analyze the cause and effect relationship of spatial change in villages and the characteristics of the change [9]. The goal is to investigate the factors that influence spatial change in villages and the specific mechanism through which they respond [10]. The second research approach involves integrating spatial analysis methods to provide a scientific explanation for the process of village spatial change. This includes applying various spatial analysis techniques [11–13] such as spatial syntax [14], fractal dimension [15], and graph theory [16,17] to examine the numerical changes in village spatial change. The objective is to uncover the pattern of village spatial development and evaluate its quality. The former approach primarily examines the changes in the spatial structure of villages, providing a comprehensive understanding of the overall spatial transformation. However, it relies on logical deduction and lacks spatial information, making it challenging to directly correlate with the actual village space. On the other hand, the latter approach focuses on deducing the development process of villages based on available data [18]. This method closely aligns with the real space but is limited by the historical information available, resulting in a restricted time frame for analysis, typically focusing on the recent decades of village development [19,20]. Due to the limitations of the study, it is challenging to encompass the entire developmental history of the village from ancient times to the current day. Consequently, it may not be feasible to provide a comprehensive explanation of the overall evolution of the village. Existing research on the evolution of spatial patterns in traditional villages typically fails to adequately consider the entire temporal aspects and unique spatial characteristics of these changes. As our comprehension of traditional village spatial patterns improves and our efforts to promote traditional village spatial construction persist, the analysis of traditional village spatial and temporal evolution processes will necessarily necessitate higher criteria.

The study chooses Gaoyi Village in Western Hunan, China, as a case study, using the "process-pattern-mechanism" analytical framework to determine the village’s development trajectory. The village space’s historical pattern is rebuilt by combining historical events and merging existing high-resolution photos. Spatial analysis techniques such as SDSEA, NNA, and SDA are used to investigate the migration of the spatial center of gravity, internal spatial dispersion, and functional transfer during the village space transformation process. These analyses enable a complete assessment of village changing characteristics and the interaction logic between villagers’ behavior and space, so enabling the current spatial development of traditional villages.

## 2. Study area

**Fig 1:**
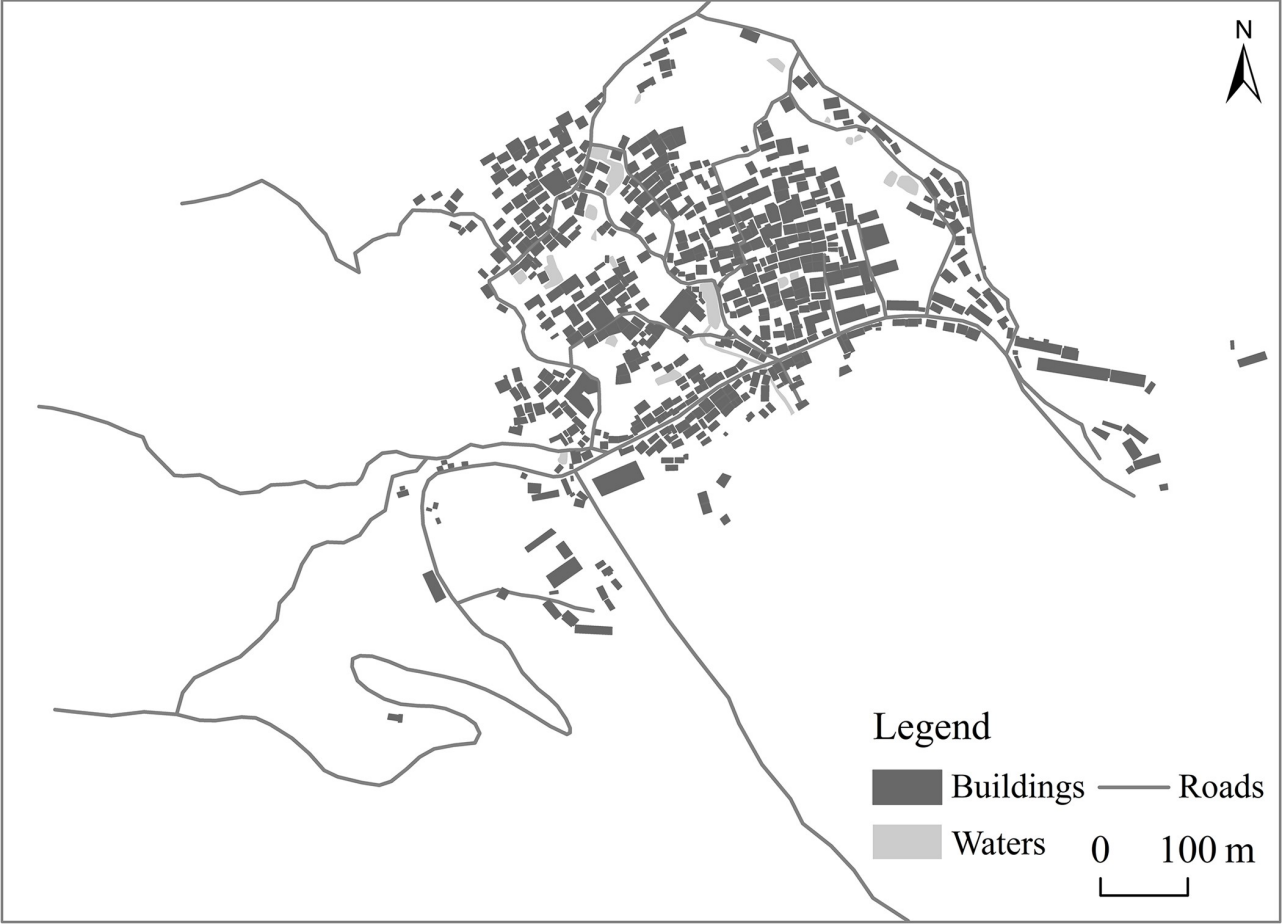
Internal spatial distribution of Gaoyi village. (The figure was drawn by the author using high resolution images and photographs taken during field research. It is similar to the original image but not identical. It is for illustrative purposes only).

Gaoyi Village, located in Western Hunan, is home to the Dong ethnic group. Surrounded by mountains on three sides and water on the other, the settlement gets its name from its resemblance to a "Taishi Chair" (An ancient Chinese seat with a backrest and armrests on both sides), which has a history of over 600 years. The village’s development has fluctuated dramatically, prospering with the advancement of land and water-based commercial transportation and diminishing as it waned. The village’s development has fluctuated dramatically, prospering with the advancement of land and water-based commercial transportation and diminishing as it waned. As a result, the village’s internal space has changed, documenting the entire development path. Interpreting the evolution of internal space can improve understanding of the village’s ancient commercial development, shed light on the potential relationship between village spatial changes and villagers’ behavior, and investigate the characteristics of spatial and temporal village space changes, as well as their driving mechanisms. Fig 1 below depicts the internal geographical distribution.

## 3. Materials and methods

### 3.1 Data sources

The data used in this study encompass textual information, survey results, high resolution images, and village historical event points. Textual sources include existing village survey books, records, genealogies, and related study literature. Survey results include information about village land types and building ages. High-resolution photos at level 20 enable Google Earth to identify village features. Village’s historical event point comprises of attribute information about historical events in the village and their locations. Table 1 details these data sources and applications. Text data are used to extract historical event information from the village’s evolution, which is then paired with high-resolution photos and survey results to determine the village’s general position and size. On-site visits and study help to thoroughly understand the history of village development, which aids in determining and validating the authenticity of extracted textual events. Finally, village historical event point is compiled, which includes records on village space age, names, spatial characteristics, functional uses, and other relevant information.

**Table 1:** Description of data sources and use.

Data category	Data sources	Description of use
Textual material	Internet http://www.dmctv.cn/, Zhu L’s *Empirical study of traditional villages in China*: *Gaoyi Village* [21] and Li QX’s *Gaoyi Village* [22], Publicity publication *Magical Village*: *Ancient Architecture of Gaoyi*.	Based on the events that occurred in the history of the village and the use of the buildings recorded in the text, the use of the internal space of the village in each period (including the existing and disappeared space) is surmised. Among them, the ritual, cultural and educational, public office and storage buildings are uniformly categorised as public space; the shops, merchant ports and moneychangers are uniformly categorised as commercial space; and the rest of the buildings are categorised as residential space.
Engineering survey information	Current building age classification charts for the Gaoyi Village plan text.	Provides information on the age of the buildings in the village and by interpreting it, it is possible to understand the period and distribution of the existing buildings. The village buildings are divided into four periods: "Ming and Qing Dynasties and before", "Republican period", "1950s-70s" and "1980s-present". "It is divided into four periods.
High Resolution Imaging	Google Earth https://earth.google.com/	For the correction of planning textual information that does not contain a spatial reference system so that it can be used properly for distance calculations as well as other spatial analyses. To update spatial changes in villages after 2015.
Village historical event point	Based on the above data and visits and research to organize.	Record historical events in the hamlet and analyze the process of spatial change in the community.

### 3.2 Methods

#### 3.2.1 Standard deviation elliptic analysis

The overall architectural center of gravity of the village, as well as the distribution of the center of gravity for each type of functional space, are identified in order to examine spatial distribution and temporal change features [23–25]. During processing, weight values are assigned to the space’s area attribute field, with a coverage of 63%. Calculated processing result values include center coordinates, long axis, short axis, ellipse flatness, and offset. Ellipse center coordinates typically represent the center of gravity of a spatial distribution; the long axis identifies the direction of data distribution; and the ellipse flatness symbolizes the range of data distribution. A shorter short axis indicates more obvious centripetal data nature, whereas a longer short axis shows more data dispersion. The difference between long and short axes, as well as their ratio, convey data directional qualities: larger flatness indicates more significant data directionality, whilst lower values indicate no directional characteristics. The difference between two distinct ellipse center coordinates represents spatial center of gravity movement.

#### 3.2.2 Nearest neighbor analysis

The internal agglomeration and dispersion features of the village space are discovered and correlated across time to investigate changes in aggregation and dispersion [25–27]. The ratio of observed and expected average distances between the village space’s center indicates distribution status, which is classified into aggregation, random, and discrete distributions. The distance determined in this research is the European geometric distance. The average observation distance (${\bar{D}}_{O}$) is calculated as the average distance between each center of interest and the location of its nearest neighboring center of interest. The expected average distance (${\bar{D}}_{E}$) is the expected nearest neighbor distance in stochastic mode. The average nearest neighbour ratio (*ANN*) is a measure of spatial distribution. It is calculated as the ratio of the observed distance to the expected distance. If the ratio is less than 1, it indicates a clustered spatial distribution, while a ratio greater than 1 indicates a dispersed spatial distribution. The z-score (z) indicates the standard deviation and is used to determine the confidence level of the results.


$${\bar{D}}_{O}=\frac{\sum_{i=1}^{n}d_{i}}{n}$$ (1)

$${\bar{D}}_{E}=\frac{0.5}{\sqrt{n/A}}$$ (2)

$$ANN=\frac{{\bar{D}}_{O}}{{\bar{D}}_{E}}$$ (3)

$$z=\frac{{\bar{D}}_{O}-{\bar{D}}_{E}}{SE}$$ (4)

$$SE=\frac{0.26136}{\sqrt{n^{2}/A}}$$ (5)

Where *d*_*i*_ is the distance between element, *i* is nearest neighboring element, *n* is the number of elements in the region, and *A* is the total area of all element envelopes.

#### 3.2.3 Source-destination analysis

**Fig 2:**
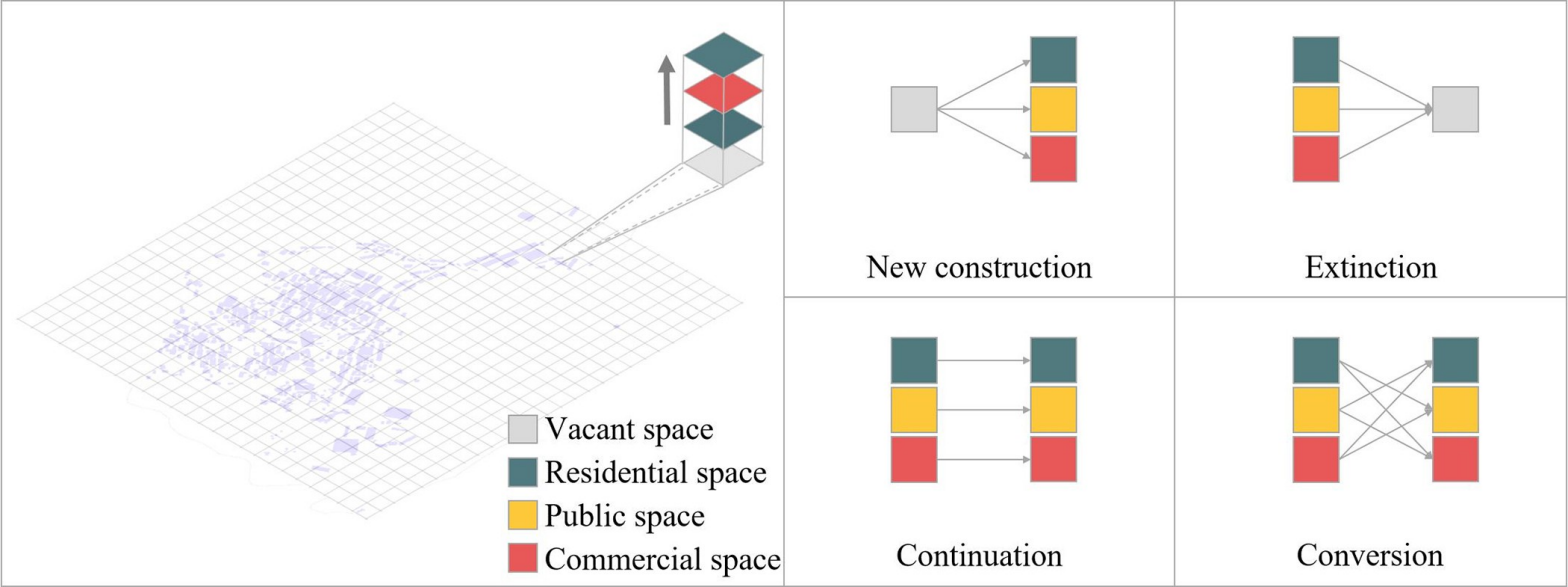
Characteristics of spatial function change in villages.

By combining the study of land use functional changes with the actual development of village space [28,29], the space is classified into four types: "residential space," "public space," "commercial space," and "vacant space." Vacant spaces are those areas where no buildings existed at some stage of the village development process. Using these four space types, a 1x1m fishing net map of the hamlet was created to investigate the functional change process using a single raster functional carrying and change. Based on the functional characteristics of individual grids in different times and village space change qualities, the process may be categorized into four categories: "new construction," "extinction," "continuation," and "conversion" (Fig 2). "New construction" refers to the process of converting a vacant space into a functional location. "Extinction" denotes a transition from a functional to a vacant space. "Continuation" refers to the grid maintaining the same functional type as in the prior era. "Conversion" implies that the two periods preceding and after the same raster are not vacant spaces; the function type is just different.

## 4. Results and discussion

### 4.1 Historical evolution of village development

**Fig 3:**
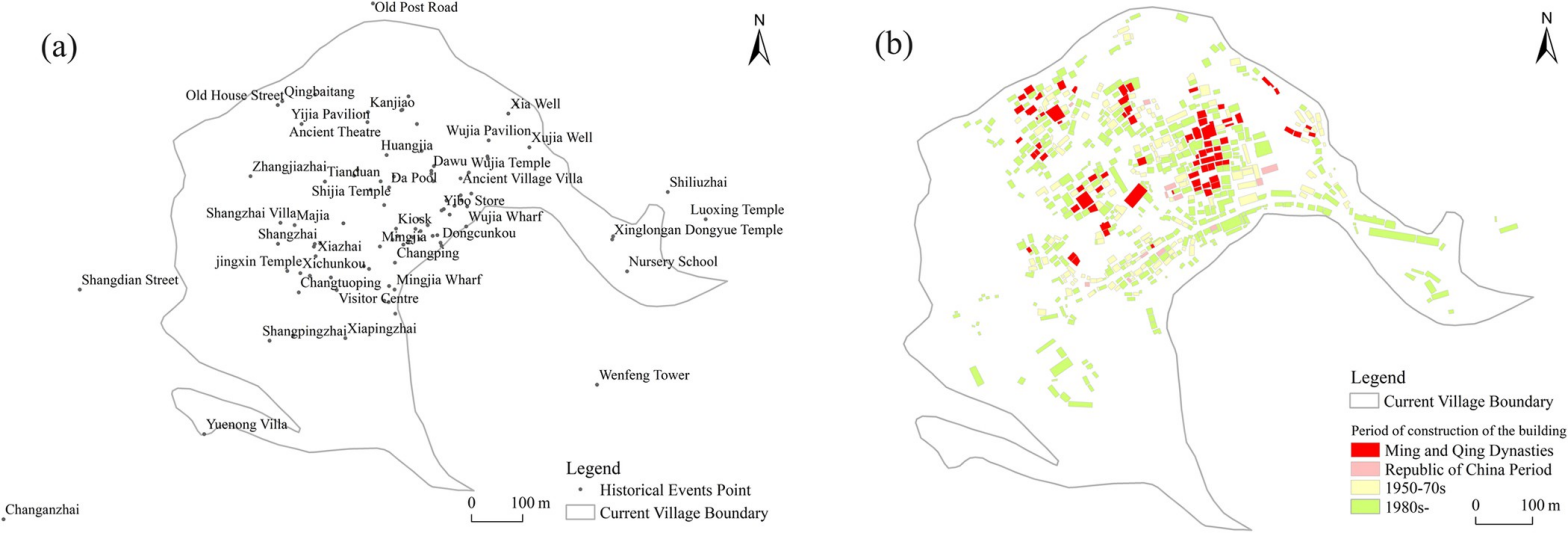
Distribution of historical event points (a) and building dates (b) in the village. (The two figures referenced the Gaoyi Village Plan text and incorporate additional information derived from the study. It is similar to the original image but not identical. It is for illustrative purposes only).

The development of the village has meant the accumulation of old spaces, some well preserved and others obliterated for later construction, making them difficult to trace. Relying simply on current building age distribution to determine each period’s village space leads to unavoidable data loss. Thus, this work first understands village history using historical materials, then spatially localizes event points by integrating them with contemporary village pictures to produce the distribution set of village historical event points (Fig 3A). Next, the collection of village event points is geographically matched with the village building age distribution vector (Fig 3B), which supplements the village’s spatial distribution in each period. The village’s development history is thus linked to historical events.

**Fig 4:**
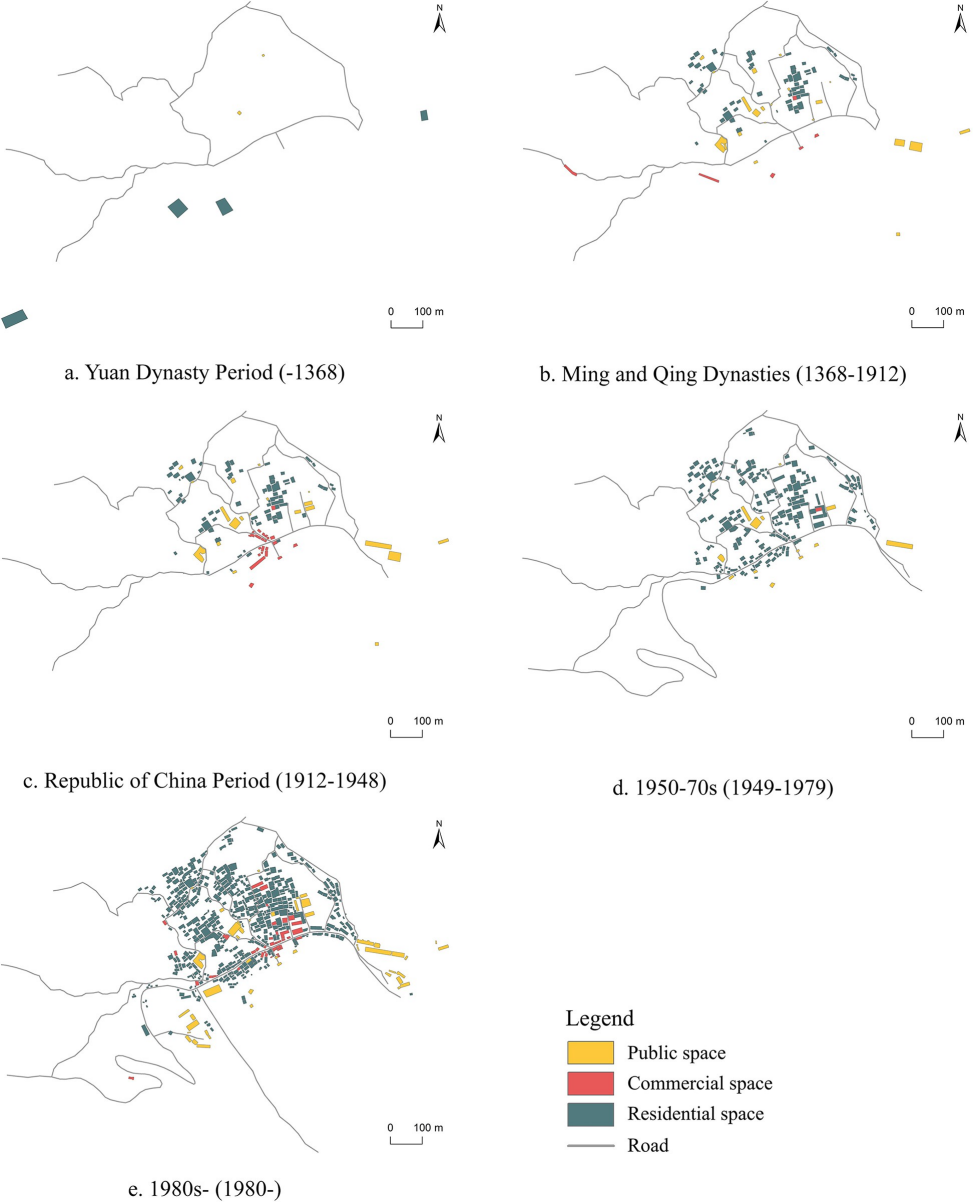
Functional zoning of the village and its change process. (The figure shows the result of putting together the Gaoyi Village Plan text, other scholars’ research [21,22], and field notes. It is similar to the original image but not identical. It is for illustrative purposes only).

Given the differences in event features, the village space is divided into three types: residential, commercial, and public, as seen in Fig 4.

Before the Yuan Dynasty, the village’s spatial distribution was dispersed, with only four primitive settlements (Changanzhai, Shangpingzhai, Xiapingzhai, and Shiliuzhai) spread across various hills, as well as two public spaces, Wutong Temple and Jiang Taijun’s Tomb, where ancient stagecoach routes facilitated trade and commerce.

During the Ming and Qing dynasties, Han generals were stationed in Gaoyi, where they established familial mansions in the village center. Following multiple surname changes, a village group dominated by the Yang surname and linked by blood ties arose. Taking use of land and sea transportation advantages, the community created its initial commercial spaces: Shangdian Street, Changtuoping, and the merchant wharf. Furthermore, public venues like ancestral halls and nunneries were built in and around the village.

During the Republic of China period, canal transportation expanded, resulting in the eventual loss of business along the historic stage road. As a result, the village’s commercial space eventually relocated to the vicinity of the pier, creating a uniform commerce polder. This move accelerated the development of commercial buildings in the surrounding space. New public spaces, such as schools, health clinics, and post offices, began to appear in the village, while residential space grew and expanded inside village groupings.

From the 1950s to the 1970s, political circumstances caused a severe shrinkage of the town’s commercial space, which was primarily used by public spaces for community groups. Regardless, village groups continued to grow. During this time, the supply and marketing association focused entirely on commercial functions, and the original "ritual" type of public space was functionally replaced. The growth of residential space was noticeable.

From the 1980s to the present, driven by modernization and the integration of culture and tourism, the village has worked to restore damaged ancestral halls and nunnery temples, improve tourist facilities such as visitor centers and parking lots, and spontaneously convert residences into lodgings for convenience. During this time, the village office building and school, as well as other public services, changed position and scale.

### 4.2 Processes and patterns of village evolution

#### 4.2.1 Characteristics of the shift in the center of gravity of village evolution

**Fig 5:**
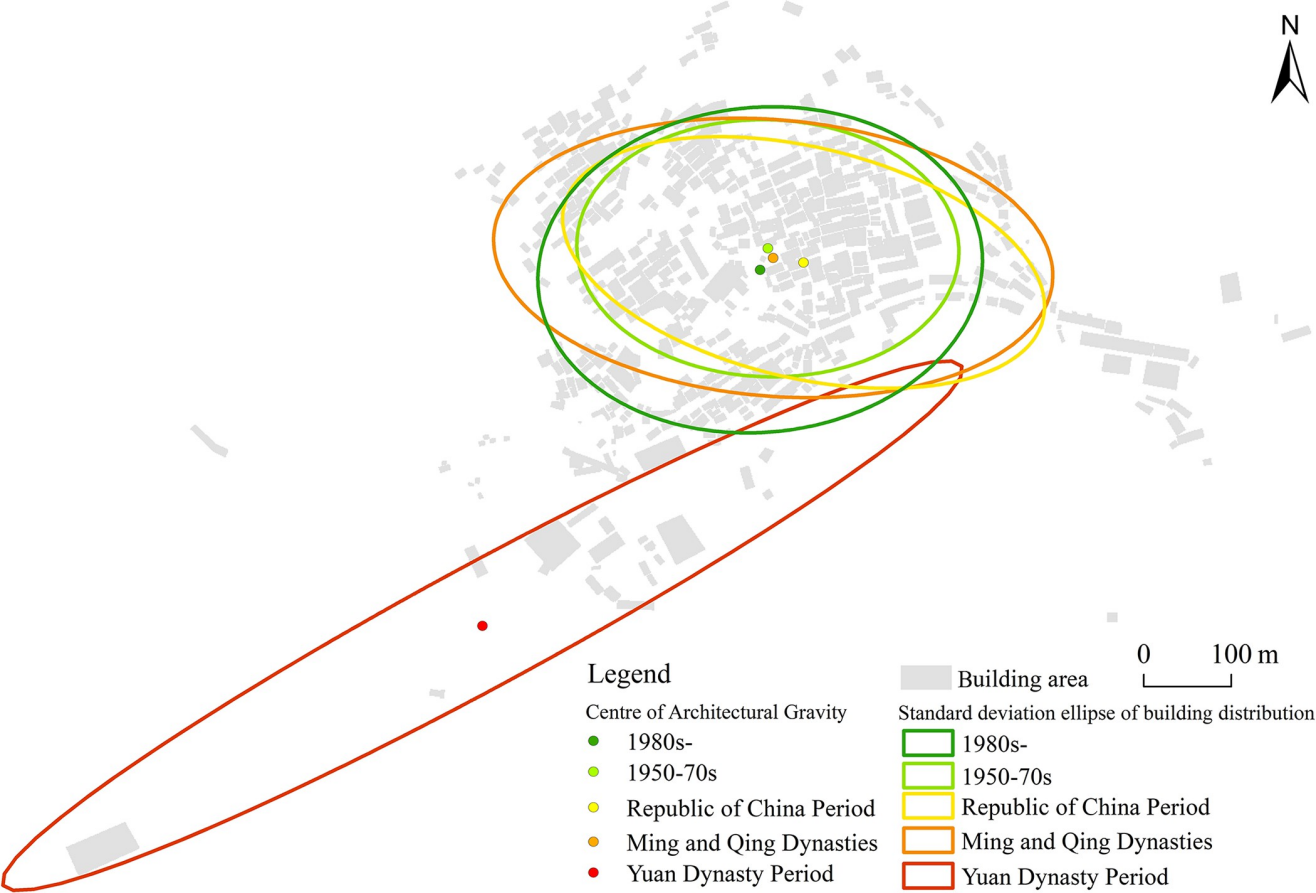
Standard deviation ellipse of the spatial distribution of villages. (The figure was created with high resolution imagery and information gathered from field research. It is similar to the original image but not identical. It is for illustrative purposes only).

The SDSEA was used to investigate the change characteristics of village spatial distribution (Fig 5). The focal point of village spatial development went from northeast (Yuan Dynasty-Mingqing Period) to southeast (Mingqing-Republic of China Period), then northwest (Republic of China to 1950s-70s Period), and lastly southwest (1950s-70s to 1980s Period). The largest movement happened during the Yuan Dynasty-Mingqing Period, showing considerable spatial construction changes between the two periods. Subsequent changes were smaller. The minor shift from the Ming-Qing dynasty to the present shows that space was stable during that time. The elliptical curvature (Yuan Dynasty: 0.11, Ming Dynasty: 0.49, Republic of China: 0.47, 1950s-70s: 0.67, 1980s-: 0.73) suggests overall spatial filling in all directions, resulting in balanced village space development.

**Fig 6:**
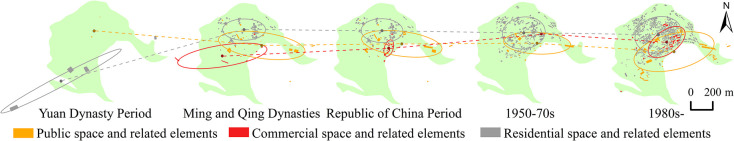
Spatial standard deviation ellipse of village function and its variation process. (There are only two public spaces in the Yuan dynasty village, Wutong Temple and Jiang Taijun’s Tomb, and only one commercial space in the 1950s-70s village, the Supply and Marketing House, both of which could not be subjected to the standard deviation ellipse treatment, and therefore their geometric centers were used as substitutes. The figure was created with high resolution imagery and information gathered from field research. It is similar to the original image but not identical. It is for illustrative purposes only).

To investigate the spatial and temporal changes in the center of gravity distribution, the SDSEA was performed for each functional region of the village in each period (Fig 6) The village’s residential space changed consistently, shifting from the surrounding hills to the central hinterland, developing distinct group layouts, and eventually filling gaps between groups. Commercial spaces first appeared during the Yuan Dynasty, developed into trading areas during the Ming and Qing Dynasties, moved from the outskirts to the centre of the village and dispersed around the village, concentrated near the wharf and its surroundings during the Republican period, replaced by the Supply and Marketing Cooperative in the 1950s-70s, later revived by cultural and tourism initiatives, and gradually integrated into the village centre.

#### 4.2.2 Characteristics of spatial dispersion of villages

The NNA was used to determine the spacing distance between each building in the village, with the goal of investigating the changing characteristics of spatial clustering degree (Table 2).

**Table 2:** Results of NNA of spatial distribution of villages by time period.

Type	*Phase*	*Average observation distance (m)*	*Expected average distance (m)*	*Nearest neighbor ratio*	*Z-score*	*Distribution type*
Space in general	-1368	307.187	153.639	1.999	4.683	Dispersed
	1368–1911	30.937	41.013	0.754	-4.557	Clustered
	1912–1949	22.766	29.237	0.779	-4.677	Clustered
	1950-70s	14.858	17.282	0.860	-4.292	Clustered
	1980s-	12.332	16.508	0.747	-12.214	Clustered
Residential space	-1368	400.627	86.364	4.639	13.923	Dispersed
	1368–1911	20.485	23.709	0.864	-2.113	Clustered
	1912–1949	20.869	24.087	0.866	-2.257	Clustered
	1950-70s	14.055	16.225	0.866	-3.972	Clustered
	1980s-	11.988	15.394	0.779	-9.936	Clustered
Public space	-1368	195.815	6.997	27.986	73.011	Dispersed
	1368–1911	70.582	62.589	1.128	1.146	Random
	1912–1949	71.323	64.062	1.113	0.994	Random
	1950-70s	83.187	59.643	1.395	2.826	Dispersed
	1980s-	34.920	46.863	0.745	-3.342	Clustered
Commercial space	-1368	-	-	-	-	-
	1368–1911	170.467	82.149	2.075	5.038	Dispersed
	1912–1949	21.656	17.689	1.224	2.057	Dispersed
	1950-70s	-	-	-	-	-
	1980s-	33.967	37.535	0.905	-1.135	Random

**Note:** There is no commercial space distribution at this time in the meta-period, and only one commercial space during the 1950s-70s, neither of which could be processed computationally.

The average and predicted observation distances of the village space have been decreasing since the Yuan Dynasty, demonstrating a general clustering tendency during village development. The nearest neighbor ratio, which measures the degree of element aggregation, was significantly more than 1 before the Yuan Dynasty, showing dispersal, but much smaller than one subsequently, indicating aggregation. The shift in nearest neighbor ratio depicts the geographical dispersion dynamics of villages as "dispersed-aggregated-slightly dispersed-slightly dispersed-aggregated again". Prior to the Yuan Dynasty, ethnic minorities occupied villages, which were generally dispersed. During the Ming and Qing dynasties, they gathered in the village center, producing a group structure with clear spatial aggregation. During the Republic of China’s 1950s and 1970s, villagers continued to grow into adjacent spaces while preserving some geographical dispersion but with an overall tendency toward aggregation. Following the 1980s, villages gradually filled the gaps between clusters, indicating a new trend of overall aggregation with a stronger internal aggregation tendency.

Residential space steadily moved from dispersed to aggregated over time, reflecting the village’s general spatial evolution. As a result, it is argued that changes in residential space are the fundamental driving force behind village spatial dynamics. Public and commercial space distributions are primarily non-aggregated, incorporated within residential space and its surroundings. However, the closeness ratio between surrounding public and commercial spaces has been dropping over time, with public space exhibiting aggregation tendencies since the 1980s. The random distribution of commercial space since the 1980s could be attributed to villagers’ autonomous conversion of houses into Homestays, which have inherent randomness.

#### 4.2.3 Characteristics of spatial area changes in villages

**Fig 7:**
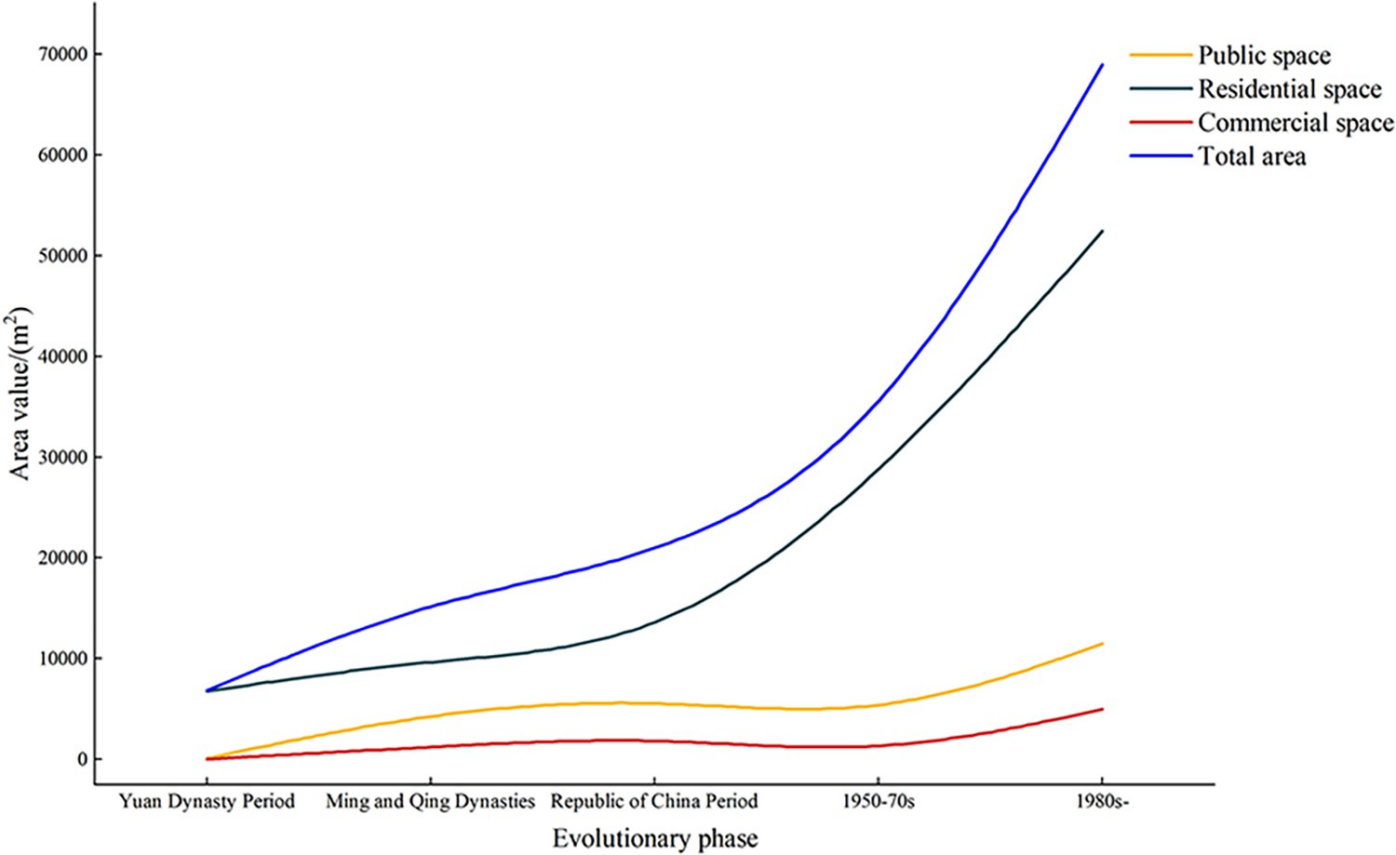
Statistics on changes in the area of each functional area of the village. (The values used for graphing have been uploaded in the form of "Supporting Information files").

The area data of village functional zones for each period show a constant spatial ratio hierarchy: residential space > public space > commercial space, with overall village internal space increasing over time (Fig 7). Notably, village expansion activities increased from the 1950-70s, whereas commercial and public spaces declined. Curves illustrating total area change and residential space change are tightly aligned, representing the fundamental components of village functional space. Similarly, the curve changes for public and commercial spaces show parallel trajectories, implying common traits or interdependence in the village’s history.

#### 4.2.4 Characteristics of spatial function transfer in villages

**Fig 8:**
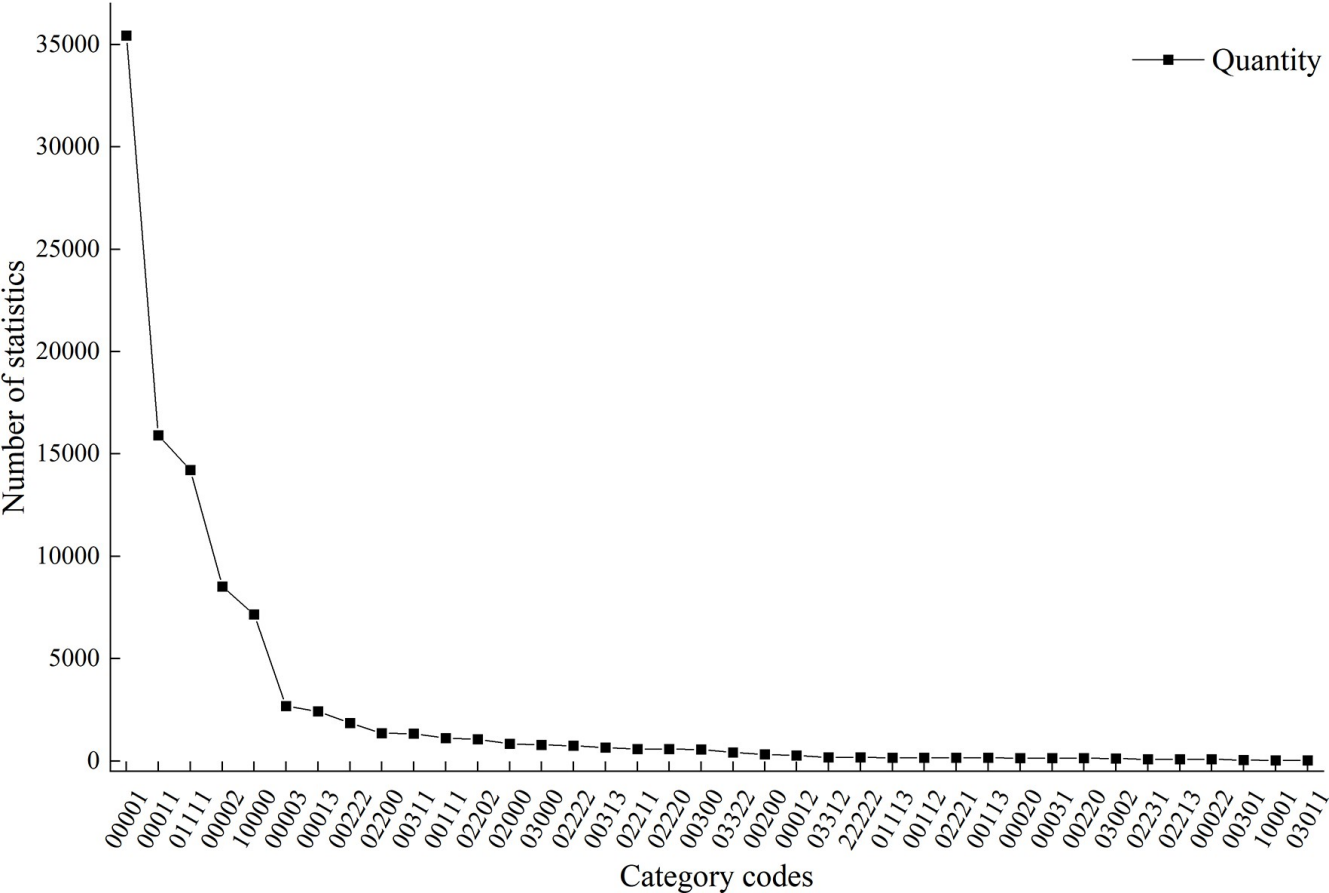
Classification and number statistics of functional change codes of village plots. (The values used for graphing have been uploaded in the form of "Supporting Information files").

The fishing net tool was used to split the hamlet into 1x1m grids classified by functional attributes: vacant space (0), residential space (1), public space (2), and commercial space (3). The analysis of functional changes in village spaces produced the following results:

The most common codes and percentages are as follows: 00001 (35.14%), 00011 (15.77%), 01111 (14.10%), 00002 (8.45%), and 10000 (7.10%). These correspond to newly constructed residential spaces after 1980, newly constructed and preserved residential spaces between 1950 and 1970, newly constructed and conserved building spaces, new public spaces after 1980, and vanished building spaces during the Yuan Dynasty (Fig 8). These data indicate that the construction and continuation of residential spaces lay the groundwork for village space expansion, with changes occurring predominantly after the 1980s.

Prior to the Republican period, the village space did not suffer direct functional transformations; the main changes were new construction and the extinction of space. During the Ming and Qing Dynasties, vacant spaces were predominantly changed into "public space" and "residential space." Residential and vacant acres were reversed from the Yuan Dynasty to the Ming and Qing Dynasties, indicating a probable significant shift in villagers’ preferred places of residence throughout this time period. Subsequent changes in function were dominated by the conversion of commercial space to residential space in the 1950-70s, and residential space to commercial space after the 1980s (Table 3).

**Table 3:** Statistics on spatial changes in villages. (The table shows the number and percentage of different types of spaces in the grid. The unit is number).

Phase	Type of space	*Continuation*	*New construction*	*Extinction*	*Conversion*			*Preserved volume*	*Changed volume*	*SCPPR %*	*STPPR %*
					*Residential*	*Public*	*Commercial*				
Yuan and Ming dynasty	Residential	-	7185	-	-	0	0	0	7358	-	-
	Public	-	173	-	0	-	0				
	Commercial	-	0	-	0	0	-				
Ming and Qing dynasties	Residential	0	14391	7185	-	0	0	173	28643	1	2
	Public	173	5524	0	0	-	0				
	Commercial	-	1543	-	0	0	-				
Republic of China	Residential	14391	1441	0	-	0	0	19857	8175	71	69
	Public	4858	2331	839	0	-	0				
	Commercial	608	2629	935	0	0	-				
1950-70s	Residential	15832	18645	0	-	675	2192	22871	22069	51	82
	Public	3665	227	2427	0	-	419				
	Commercial	0	144	626	0	88	-				
1980s-	Residential	33209	35525	0	-	156	232	41017	48794	46	91
	Public	3285	9707	870	628	-	-				
	Commercial	0	2692	0	3507	0	-				

**Note:** Preserved volume = Continuation + Conversion; Changed volume = New construction + Extinction; Spatial cis-period preservation rate (SCPPR) = Preserved volume / (Preserved volume + Changed volume); Spatial trans-period preservation rate (STPPR) = Preserved volume / (Preserved volume_(t-1)_ + Changed volume_(t-1)_).

The amount of continuation and conversion was used to measure spatial stability for each time, while new construction and extinction were used to examine spatial variability. The SCPPR from the Yuan Dynasty to the Ming and Qing Dynasties was only 1%, indicating that the spatial utilization of the villages changed dramatically during the period. After the Ming and Qing Dynasties, the village SCPPR decreases sequentially, indicating that the trend of changing village space is still incremental. Meanwhile, the STPPR of the villages in the period of the Ming and Qing Dynasties has gradually increased, which means that the villages have to a great extent expanded and changed outward on the basis of the original space.

### 4.3 Analysis of village evolution and villagers’ behavioral mechanisms

#### 4.3.1 Characterisation of the spatial evolution of villages

Gaoyi Village has transitioned from traditional farming to ancient trading, modern agricultural, and cultural and tourism development. By examining the geographical distribution and functional transfer characteristics of Gaoyi Village, the spatial conditions at its five-time nodes are analyzed, allowing the village’s developmental stages to be refined. These five-time nodes represent the stages of commerce and trade emergence, prosperity, decline, shift in production, and cultural and tourism growth.

The budding period of commerce: compared to the Yuan Dynasty and prior, village space usage is limited, residential spaces are the primary component, and the overall functional organization is relatively single and spatially separated.

Prosperous period of trade and commerce: during the Ming and Qing periods, all functional sections of the village experienced growth, with the residential and public spaces showing the most noticeable increase. Because of the benefits of land and water transportation, the village’s commerce grew quickly, and merchants congregated in trading spaces such as "Shangdian" street and "Changtouping", as well as commercial wharves and other sites utilized for water transportation and trade.

Decline of commerce: during the Republic of China period, the decrease of commerce on land routes resulted in the movement of commercial spaces traditionally associated with the routes to the wharves. With the migration of the relevant departments from Gaoyi to Hongjiang, the number of merchants who remained in the village reduced, as did demand for commerce in the village, resulting in additional commercial space contraction and a more concentrated distribution pattern.

The period of production change: during the 1950-70s, inspired by macroeconomic policies, the commercial space in the village was significantly reduced and transformed into residential and public services. The communal system fully prohibited private trade, with the supply and marketing society solely responsible for meeting commodities supply demands. While many public spaces preserved their public character, their use shifted, and several public buildings were removed during this time. Residential spaces grew during the transformation process, whereas commercial and public spaces contracted.

Cultural and Tourism Construction Period: from the 1980s to the present, the growth of cultural and tourism integration has increased the need for village construction, resulting in the restoration and enlargement of the original public and commercial spaces. Businesses such as shops and hostels, private museums for viewing, and tourism-supporting amenities such as parking lots and visitor centers have developed throughout the village, integrating into its spatial fabric and complicating the hamlet’s functional structure.

#### 4.3.2 Mechanisms of spatial evolution based on villagers’ behaviour

**Fig 9:**
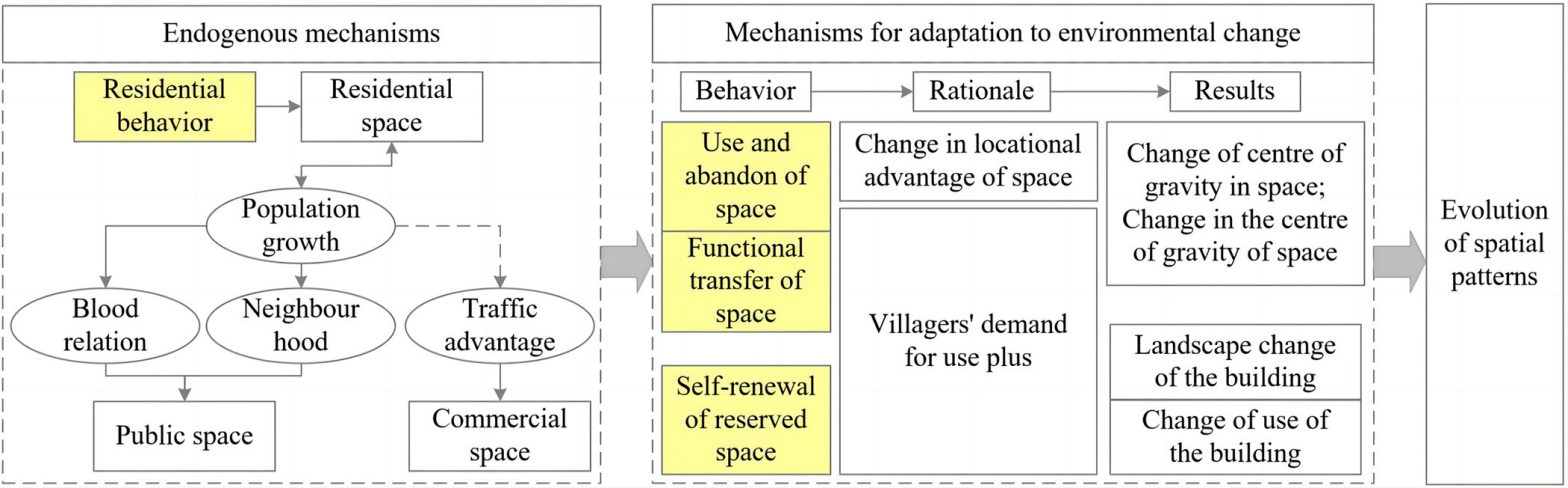
Mechanisms of "behavioral-spatial" change among villagers.

The village space is inextricably related to the conduct of its residents, with the villagers’ initiatives playing a central role in its construction. The evolution of village space represents the villagers’ representation of their needs, as well as changes to the spatial arrangement in response to natural and social contexts. The study examines three aspects of village activity: residential behavior, new construction, abandonment and functional transfer of space, and self-renewal of preserved space (Fig 9).

*The residential behavior of villagers is the foundation of village space development and construction*. Villagers’ residential behavior and the village’s residential space are inextricably intertwined. Residential space has played a vital role in the evolution of village spatial distribution, dispersal, and relocation, implying that villagers’ residential behavior serves as the foundation for village space. Initially, humans from other spaces migrated to this site, creating residential spaces. With population growth and progressive reproduction, the need for housing rises, necessitating the expansion of residential spaces. As the population grows, social networks such as blood relatives and neighbors form, resulting in the building of public spaces like ancestral halls, pavilions, and schools.

Meanwhile, if the village has transportation advantages, such as proximity to ancient stagecoach routes and waterways, it promotes the exchange of products, resulting in the formation of official venues for commerce and trade inside the community. A settled population also increases the security of commercial space, resulting in a positive feedback loop that supports the development of commercial space within the village. This demonstrates how villagers’ residential behavior drives village development, imbuing the village space with boundless possibilities.

It also means that concerns such as the hollowing out of villages in modern times can be linked to the loss of village populations and the resulting lack of development momentum. While modern protection techniques such as relocation and off-site preservation for traditional villages attempt to reduce human-caused harm and preserve the original village appearance, they may also stall local spatial development.

*Using*, *abandoning*, *and transferring functions may assist to evolve village space*. The use and abandonment of space are directly evident in the construction and demolition of village buildings. For instance, in the Yuan Dynasty period, the integration of the Han Chinese led to a shift in the main body’s choice of settlement sites, with original hilltop settlements losing their advantages and being abandoned, leaving only ruins as evidence. Population growth spurred increased demand for housing, resulting in ongoing construction of new houses in the village. During the Ming and Qing dynasties, the demand for new houses rose due to improved land and water transportation. However, trading places like "Shangdian Street" and "Changtuoping," established during the Ming and Qing Dynasties to leverage the commerce and transportation advantages of ancient stagecoach routes, gradually disappeared as the functionality of these routes declined.

Functional space transfer involves relocating functions from their original location within the village or repurposing the original space with new functions. This transfer was primarily observed after the Republic of China period, with notable activity in government office and cultural and educational spaces within public spaces. For instance, in the 1980s, the government office, originally situated near the Yijia Ancestral Hall, was relocated to the southwestern side of the village to establish a new office area, transforming the original site into a plaza after demolition. Similarly, cultural and educational spaces underwent a transformation from private schools to public schools, ultimately transitioning back to residential spaces. These spaces experienced a shift from village residences to independent school buildings before relocating to the southeast side of the village to form a dedicated cultural and educational area. Notably, the trend of repurposing original spaces with new functions is particularly evident in the construction of homestays, where villagers adapt original residential spaces to meet the accommodation needs of tourists, driven by the development of cultural tourism.

Generally, space abandonment and function transfer manifest as villagers selectively adjusting the village’s internal space based on environmental changes, following a principle of "eliminating the best and the worst," ultimately enhancing the village’s adaptability to its surroundings. This process will change the spatial centre of gravity and dispersion characteristics of the village.

The self-renewal behavior of spaces retaining their original functions promotes the adaptive adjustment of the village’s spatial structure. Some spaces retain their original functions amidst functional changes, as evident from the STPPR in Table 3, with the area of such spaces gradually increasing over time. However, these spaces are not entirely stable; they undergo changes in style and use influenced by villagers’ self-renewal behavior to meet evolving needs. For instance, influenced by Western architectural styles, villagers transformed traditional buildings like Yueguanglou into new-style houses. In the 1950s, during the People’s Communalization Movement, the original public space of ancestral halls in the village was repurposed into public space for offices and warehouses of a public service nature. In short, changes in residential space primarily respond to villagers’ personal needs and are driven more by individual behavior, whereas changes in public space reflect broader village demands during specific periods, showcasing characteristics of the times.

## 5. Conclusions

In this paper, the historical spatial conditions of villages are reconstructed by integrating historical events of village development with the current spatial distribution of villages. Through the comprehensive application of various spatial analysis methods, it examines the characteristics of spatial changes in the village, thereby analyzing the disparities in spatial conditions at different stages of village development and the mechanisms behind villagers’ behaviors. The conclusions drawn are as follows:

1) The village space can be classified into three main functional zones: "residential," "public," and "commercial". The distribution of area shares indicates that the residential space > public space > commercial space at each stage. Among these, residential space holds a predominant position in the evolution of village space, with villagers’ residential behavior serving as the driving force behind its construction and development. Public space represents the social network relationships formed through the growth of the village population, reflecting the collective spatial needs of the residents. Commercial space emerges from villagers’ entrepreneurial activities, primarily stemming from their spontaneous actions.

2) The evolution of the spatial function of the village is a process in which the internal elements gather with each other and the way of converting the internal function changes from simple to complex. The center of gravity of various types of village space distribution has changed from being far away from each other to being close to each other, and the internal space has shown a distribution pattern from being discrete to being aggregated, and the village space as a whole has developed towards the trend of aggregation. Functional changes in village space have gradually developed into a new form of mutual transformation of functions from new construction and extinction in the early days, and the form of functional changes in the internal space of villages has become more complicated.

3) As the functional transformation of village space accelerates, the preserved functional space within the village gradually expands. Under the mutual effect of maintained and altered spaces, the village space as a whole evolves toward a self-adjustment route that is in line with the current social environment and the villagers’ genuine requirements. In other words, conformance with the current social environment and the villagers’ aspirations is the primary direction of village space evolution.

This paper takes Gaoyi Village as a case study and finds that Gaoyi Village as a whole has experienced the continuous growth of total spatial area and the retention and change of functional location. It can be taken as a typical traditional village developed due to ancient commercial transport, reflecting the general phenomenon of the spatial evolution of traditional villages in the Western Hunan region. At the same time, it can also be compared with other villages in non-Western Hunan regions as a way to reveal certain common characteristics of village evolution that are not restricted by geographical regions. For example, the spatial evolution of Xijiao Village in Shanxi Province, analysed by Lin ZR [8], is similar to that of Gaoyi Village analysed in this paper. In the early stage of village construction, the distribution was relatively scattered, and experienced a series of spatial evolution from dispersion to aggregation and then to expansion. While this paper delves into the correlation between the evolution of spatial form and villagers’ behavior, it remains primarily theoretical, explaining only generalized trends in evolution without accurately quantifying the extent of spatial change in villages and the strength of its correlation with villagers’ behavior. The complexity inherent to villages entails that their morphological changes are influenced by numerous factors. Consequently, relying solely on the functional changes of village space may provide only a rough understanding of the overall process of village spatial changes, leaving the exact relationship between the two subject to inference. Furthermore, limitations in the available data pose additional challenges. Village image data typically dates back to the 1950s and 1960s at best, insufficient to comprehensively cover the entire development process of villages to date. Moreover, most village buildings lack records of their construction dates, making it feasible only to estimate their age approximately. Consequently, it becomes challenging to precisely determine the duration of intervals between different periods in village history, hindering accurate measurement of their changing characteristics.

In future research, a combination of village records, genealogies, and research interviews could be employed to refine the subcategories of village spatial functions, such as residential, public, and commercial spaces. This would serve to enhance our understanding of the process of village spatial changes. Furthermore, dividing the stages of village evolution based on existing segments would allow for a more nuanced analysis, facilitating quantification of the degree and efficiency of village changes using methods such as "amount of spatial changes / time". Additionally, the advantage of remote sensing monitoring in the protection of historical monuments [30] allows for the identification of a number of villages with similar geographic environments or similar development processes as the case study villages in this paper. These can be quickly and widely identified as samples for analysis. Through the horizontal comparison of multiple villages, the vacant contents of each other’s historical development records are confirmed. This approach would logically supplement the spatial evolution process of villages, leading to a more comprehensive understanding of their spatial morphology and evolutionary mechanisms over time.

This paper is based on spatial changes alone. However, in light of the recent decline in the birth rate in China, it is possible that the village population may also reach its peak at this time. From the perspective of self-renewal of traditional villages, the factor of "population growth" has the greatest influence on the expansion of village space. However, this factor will not be able to support the further expansion of villages, and the residential space may stop developing in terms of area. Furthermore, public service spaces such as primary and middle schools related to children’s education may shrink inwards or merge with other places and disappear from the village. It is possible that commercial space may gradually annex part of the residential and public space under the promotion of rural tourism and rural recreation, becoming the most active factor in the future spatial change of the village and changing the original proportion of the three types of space in the village. However, this is only a personal speculation, and further analysis of the detailed data is required before a final conclusion can be reached on the specific changes to the village space in the future.

## Supporting information

S1 File (DOCX)

## References

[1] Tang HL, Li N. Analysis of Spatial Form and Pedigree Construction of Traditional Villages in Western Hunan. Chinese & Overseas Architecture. 2024; 30(1): 49–54. doi: 10.19940/j.cnki.1008-0422.2024.01.009

[2] Zhang SW, Fang Y, Wu DH, Shen K. Transformation of Cultural Landscape in Traditional Villages in the Guangfu Area in South China: Evolution, Alienation, and Mutation. Heritage Architecture. 2022; 7(3): 21–30. doi: 10.19673/j.cnki.ha.2022.03.003

[3] Liu X, Li Y, Wu Y, Li C. The Spatial Pedigree in Traditional Villages under the Perspective of Urban Regeneration: Taking 728 Villages in Jiangnan Region, China as Cases. Land. 2022; 11(9), 1561. 10.3390/land11091561.

[4] Deng YY, FU XX, Zheng WW, Zhang HB. Representation, measurement and attribution of spatial order of traditional villages in southern Hunan. Geographical Research. 2021; 40(10): 2722–2742. doi: 10.11821/dlyj020210651

[5] Fu J, Zhou JL, Deng YY. Heritage values of ancient vernacular residences in traditional villages in Western Hunan, China: Spatial patterns and influencing factors. Building and Environment. 2021; 188: 107473. 10.1016/j.buildenv.2020.107473.

[6] Huang X, Gu Y. Revisiting the spatial form of traditional villages in Chaoshan, China. Open House International. 2020; 45(3): 297–311. 10.1108/OHI-05-2020-0027.

[7] Qin QH, Xiao DW, Huang SX, Tao J, Zeng Y. Integration, Differentiation and Mutation: Evolution of Spatial Types of Ancient Zhangzhou Traditional Villages. Architectural Journal. 2021; 68(S1): 1–6.

[8] Lin ZR, Zhang JP, Zhang X, Ding ZH. Study on the Evolution of Spatial Form of Business-Oriented Traditional Villages in Jingxing Ancient Road: A Case Study of Xijiao Village. Pingding County, Shanxi Pingding County, Shanxi. Modern Urban Research. 2019; 34(9): 10–16. doi: 10.3969/j.issn.1009-6000.2019.09.002

[9] Chen Y, Li JJ. Analysis of Spatial Form Evolution and Traditional Architecture Features of Zhoufang Historical Village from the View of “Blood-industry Relationship”. Housing Science. 2023; 3(8): 38–44+57. doi: 10.13626/j.cnki.hs.2023.08.008

[10] Yang R, Lu JF, Li W. Evolution and Influential Mechanism of Multi-dimensional Space of Typical Traditional Villages in the Metropolitan Fringe of Pearl River Delta. Economic Geography. 2022; 42(3): 190–199. doi: 10.15957/j.cnki.jjdl.2022.03.020

[11] Li YB, Liu YX, Luo GJ. The Multiple Paths of the Rural Settlement Evolution in the Peakcluster-depression Area of Central Guizhou Province: A Case Study in Houzhaihe, Puding County. Journal of Natural Resources. 2018; 33(1): 99–113.

[12] Xi JC, Wang XG, Kong QQ, Zhang N. Spatial morphology evolution of rural settlements induced by tourism: A comparative study of three villages in Yesanpo tourism area, China. Acta Geographica Sinica. 2014; 69(4): 531–540. doi: 10.11821/dlxb201404009

[13] Lin Z, Liang Y, Liu X. Study on spatial form evolution of traditional villages in Jiuguan under the influence of historic transportation network. Heritage Science. 2024; 12(1), 29. doi: 10.1186/s40494-024-01153-0

[14] Chen C, Li BH, Yuan JL, Yu W. Spatial Morphology Cognition of Traditional Village Based on Space Syntax: A Case Study of Qinchuan Village of Hangzhou. Economic Geography. 2018; 38(10): 234–240. doi: 10.15957/j.cnki.jjdl.2018.10.028

[15] Du J, Hua C, Yu YF. Research on Spatial Evolution of Traditional Settlements: The Case Study of Qianzhong Tunpu. Urban Development Studies. 2017; 24(2): 47–53. doi: 10.3969/j.issn.1006-3862.2017.02.007

[16] Song JH, Hu YX, Gong XD. Revealing the Spatial Structure Evolution Law of Hong Village with Social Network Analysis Method. Urbanism and Architecture. 2020; 17(10): 100–105. doi: 10.3969/j.issn.1673-0232.2020.10.020

[17] Jin LC, Jiao S. A Graph Theory Based Study on the Structure of Public Space in Traditional Villages. Planners. 2019; 35(2): 52–57. doi: 10.3969/j.issn.1006-0022.2019.02.008

[18] Yang X, Pu F. Cellular automata for studying historical spatial process of traditional settlements based on gaussian mixture model: a case study of Qiaoxiang village in Southern China. International Journal of Architectural Heritage. 2018; 10.1080/15583058.2018.1553077.

[19] Gao WT, Li XY, Liu LR. Landscape Pattern and Ecosystem Service Value Evolution of Traditional Villages in the Landscape: Taking Jiuxian Village of Guilin as an Example. Development of Small Cities & Towns. 2024; 42(2): 67–75.

[20] Zhou ZX, Wang XD, Liu JW, Liu SM, Luo YW. Study on the Spatial Evolution Features of the Mountainous Villages: A Case of Baishui River Valley, Central Guizhou. Urban Development Studies. 2018; 25(7): 97–105. doi: 10.3969/j.issn.1006-3862.2018.07.014

[21] Zhu L. Empirical study of traditional villages in China: Gaoyi Village. 1st ed. Changsha: Zhongnan University Press; 2019.

[22] Li QX. Gaoyi Village. 1st ed. Beijing: Tsinghua University Press; 2010.

[23] Zhang D, Shi Z, Cheng M. A Study on the Spatial Pattern of Traditional Villages from the Perspective of Courtyard House Distribution. Buildings. 2023; 13(8): 1913. 10.3390/buildings13081913.

[24] Wu C, Chen M, Zhou L, Liang X, Wang W. Identifying the spatiotemporal patterns of traditional villages in China: a multiscale perspective. Land. 2020; 9(11): 449. 10.3390/land9110449.

[25] Li T, Li C, Zhang R, Cong Z, Mao Y. Spatial heterogeneity and influence factors of traditional villages in the Wuling Mountain area, Hunan Province, China based on Multiscale Geographically Weighted Regression. Buildings. 2023; 13(2): 294. 10.3390/buildings13020294.

[26] Mitchell A. The ESRI Guide to GIS Analysis. Volume 2. Redlands: ESRI Press; 2005. ISBN 978–1589481169.

[27] Li Y, Du J, Ran D, Yi C. Spatio-temporal distribution and evolution of the Tujia traditional settlements in China. Plos one. 2024; 19(3): e0299073. doi: 10.1371/journal.pone.0299073 . 38466756 PMC10927083

[28] Xi J, Zhao M, Ge Q, Kong Q. Changes in land use of a village driven by over 25 years of tourism: The case of Gougezhuang village, China. Land Use Policy. 2014; 40: 119–130. 10.1016/j.landusepol.2013.11.014.

[29] Käyhkö N, Fagerholm N, Asseid B S, Mzee A J. Dynamic land use and land cover changes and their effect on forest resources in a coastal village of Matemwe, Zanzibar, Tanzania. Land Use Policy. 2011; 28(1): 26–37. 10.1016/j.landusepol.2010.04.006.

[30] Guo H, Chen F, Tang Y, Ding Y, Chen M, Zhou W, et al. Progress toward the sustainable development of world cultural heritage sites facing land-cover changes. The Innovation. 2023; 4(5). doi: 10.1016/j.xinn.2023.100496 37663934 PMC10472305

