# Supplementary material for: Analysis of the evolutionary process of traditional village spatial pattern: The case of Gaoyi village in Western Hunan, China
Source: PLoS One. 2024 Sep 6;19(9):e0309814. doi: 10.1371/journal.pone.0309814 (PMC11379167; doi:10.1371/journal.pone.0309814)
Supplement: S1 File — (DOCX) [file pone.0309814.s001.docx]

**Statistical table of spatial types of villages in different phases.** The table is used to support the plotting of Fig 7.

| Phase | Public space (m2) | Residential space (m2) | Commercial space (m2) | Total area (m2) |
| --- | --- | --- | --- | --- |
| Yuan Dynasty Period | 125 | 6739 | 0 | 6864 |
| Ming and Qing Dynasties | 4800 | 10036 | 1237 | 16073 |
| Republic of China Period | 6163 | 11048 | 2404 | 19615 |
| 1950-70s | 3681 | 27315 | 204 | 31200 |
| 1980s- | 11513 | 52399 | 4982 | 68895 |

**Detailed Quantitative Statistics of Functional Change Codes for Village Parcels.** The table is used to support the plotting of Fig 8.

| Sequences | Category codes | Number of statistics | Sequences | Category codes | Number of statistics |
| --- | --- | --- | --- | --- | --- |
| 1 | 00001 | 35441 | 20 | 03322 | 419 |
| 2 | 00011 | 15907 | 21 | 00200 | 334 |
| 3 | 01111 | 14223 | 22 | 00012 | 276 |
| 4 | 00002 | 8526 | 23 | 03312 | 189 |
| 5 | 10000 | 7159 | 24 | 22222 | 173 |
| 6 | 00003 | 2692 | 25 | 01113 | 168 |
| 7 | 00013 | 2437 | 26 | 00112 | 163 |
| 8 | 00222 | 1853 | 27 | 02221 | 156 |
| 9 | 02200 | 1364 | 28 | 00113 | 154 |
| 10 | 00311 | 1342 | 29 | 00020 | 144 |
| 11 | 00111 | 1124 | 30 | 00031 | 144 |
| 12 | 02202 | 1063 | 31 | 00220 | 144 |
| 13 | 02000 | 839 | 32 | 03002 | 118 |
| 14 | 03000 | 792 | 33 | 02231 | 88 |
| 15 | 02222 | 757 | 34 | 02213 | 87 |
| 16 | 00313 | 661 | 35 | 00022 | 83 |
| 17 | 02211 | 588 | 36 | 00301 | 58 |
| 18 | 02220 | 582 | 37 | 10001 | 26 |
| 19 | 00300 | 568 | 38 | 03011 | 25 |
